# Design and evaluation of an educational and self-care application for infertile men: perspective of physician and patients

**DOI:** 10.1186/s12913-025-12900-9

**Published:** 2025-05-19

**Authors:** Jalal Nikukaran, Alireza Fallahzadeh, Mahsa Kaffashpour yazdi, Seyed Ali Fatemi Aghda, Hossein Torkashvand, Reza Akbarzade, Meysam Fallahnezhad, Hadi karimi aqda, Rahime Tajvidi Asr, Shadi Hazhir

**Affiliations:** 1 Research Center for Health Technology Assessment and Medical Informatics, School of Public Health, Shahid Sadoughi University of Medical Sciences, Yazd, Iran; 2 Department of Medical Library and Information Sciences, School of Management and Medical Information Sciences, Isfahan University of Medical Sciences, Isfahan, Iran; 3 Genetic and Environmental Adventures Research Center, School of Abarkouh Medical Sciences, Shahid Sadoughi University of Medical Sciences, Yazd, Iran; 4 Department of Accounting, National University of Skills (NUS), Tehran, Iran; 5 Fakher Mechatronic Research Center, Kerman University of Medical Sciences, Kerman, Iran; 6 Student Research Committee, School of Health Management and Information Sciences, Iran University of Medical Sciences, Tehran, Iran; 7 Department of Anatomical Science, School of Medicine, Iran University of Medical Sciences, Tehran, Iran; 8 Fertility and Infertility Research Center, Hamadan University of Medical Sciences, Hamadan, Iran; 9 Student Research Committee, Department of Medical-Surgical Nursing, School of Nursing and Midwifery, Iran University of Medical Sciences, Tehran, Iran; 10 Department of Educational Sciences, Farhangian University, P.O.BOX 14665-886, Tehran, Iran; 11 Health and Biomedical Informatics Research Center, Urmia University of Medical Sciences, Urmia, Iran; 12 Department of Health Information Technology and Management, School of Allied Medical Sciences, Shahid Beheshti University of Medical Sciences, Tehran, Iran

**Keywords:** Infertility, Mobile health, Self-care, Minimum data set, Assisted reproductive techniques, Needs assessment

## Abstract

**Background:**

Male infertility has played an important role in childbearing in developing countries, including Iran. With the increasing use of mobile health and the need for self-care, this study aimed to design and evaluate a mobile-based application for infertile men.

**Methods:**

In this quantitative research, a needs assessment for the content of the application was conducted using a questionnaire and the participation of 20 physicians and 60 patients. After the initial implementation of the application, a usability evaluation was conducted for 200 participants using a Questionnaire of User Interface Satisfaction (QUIS) questionnaire.

**Results:**

In this study, the needs assessment included 8 sections: Patient information, disease-related data, Nutrition, treatments and medications, physical activities, personal habits and behaviors, sexual history, software features. In the educational content section, all sub-items of section “Disease-Related Data, Sexual History” were considered “essential” by both participant groups. In this study, the views of physicians and patients differed in " Allergy to a certain food, tea consumption, Traditional medicine amount of exercise during the day, suggested time for physical activities, sleep, Consumption of alcoholic”. The results of the usability evaluation with the QUIS questionnaire, an average of 7.97 (out of 9), indicating a “good” level.

**Conclusion:**

The designed mobile application was aimed at utilizing mobile health in male infertility. With the increasing use of mobile health, it is expected that this application will enhance education, self-care, and improve the quality of life for patients. However, further investigation of its effectiveness and impact in research community and a larger sample size is needed for result generalization.

**Supplementary Information:**

The online version contains supplementary material available at 10.1186/s12913-025-12900-9.

## Background

Infertility is defined as not being able to conceive after 12 months of unprotected intercourse [1]. Infertility is a critical public health issue that is escalating globally. According to the European Society of Human Reproduction and Embryology (ESHRE) report, one in six couples worldwide experiences infertility [2]. Different studies report varying prevalence rates, ranging from 5 to 30% of couples affected, with approximately 10–15% of couples being affected in Iran [3].

Infertility is recognized as a public health problem and in most studies, the role and factors of women and men in infertility are equal. In the world, 12% of men have experienced infertility and it is considered a public health concern [4]. Most men desire to become fathers. However, men are more likely to engage in risky behaviors such as unhealthy eating, smoking, and substance abuse [5, 6] and are more exposed to harsh environmental conditions (toxins, cell phone radiation, etc.) due to their occupational conditions. However, some factors are preventable and by increasing awareness of potential factors affecting infertility (modifiable factors), the risk and need for treatment can be reduced. In general, infertility cannot be completely prevented [7, 8]. Men are less likely to seek health information and have lower health information levels than women, and information seeking is perceived as a threat to a man’s sense of masculinity and as a sign of dependency and weakness, which leads to vulnerability and commitment. Therefore, there is a need to increase the knowledge of infertile men with new educational methods, taking into account the personality and spirit of men [1, 9–11].

Mobile phone usage has been steadily increasing worldwide, with approximately 85% of youths and adults reported to use mobile phones in the United States [12, 13]. Various methods exist for educating and raising awareness among members of society and patients. Today, given the prominence of new technologies such as mobile phones, mHealth (mobile health) is considered a modern and potentially effective intervention strategy for initiating behavioral changes and providing education in the field of healthcare [14, 15]. Numerous studies [1, 16–19] indicate that mHealth, tailored to the diverse needs of individuals, is being designed as an accessible solution to address the informational requirements of the community. Furthermore, studies [20–22] have shown that both men and women are increasingly attentive to lifestyle applications and related information. On the other hand, Considering the behavioral and personality characteristics of men, their level of education is often low and it is difficult for them to ask for help, so there is a great need for general and specialized education for men in the field of infertility [23].

The results of a study showed that the participating men were only able to identify half of their infertility risk factors [24]. In another study, they developed an application called “Infotility XY” with the aim of increasing men’s awareness. 75% of men used the application and most of them considered the application very suitable for increasing fertility knowledge and were satisfied with it [25]. In another study, 10 applications in the field of male infertility were reviewed. The results showed that the educational content of the applications was not of good quality and the content needed to be improved. Also, due to the lack of participation of healthcare providers and various stakeholders in the design of the application, adherence to the guidelines was at a low level and there is a need to use the participation of various stakeholders in their design as much as possible [26].

Considering the prevalence of infertility and its impact on the lives of couples and their social circles (relatives, friends, acquaintances, etc.), the need for planning and education is more crucial than ever before [27, 28]. The global proliferation of mobile phone usage and the corresponding mobile applications have led to the utilization of mHealth to bridge the knowledge gap.

According to the best our knowledge In Iran, high-quality educational information about male infertility has been lacking [29, 30], causing concerns for couples and policymakers, as well as healthcare providers. Additionally, due to the high costs of infertility treatments and the need for population growth, the role of self-care among patients has increased. This study was conducted to overcome these challenges, increase awareness, and knowledge about male infertility. The necessary educational materials for infertile men were prepared and made available to users through a mobile phone-based application. The usability of the application was also evaluated.

## Method

**Fig. 1:**
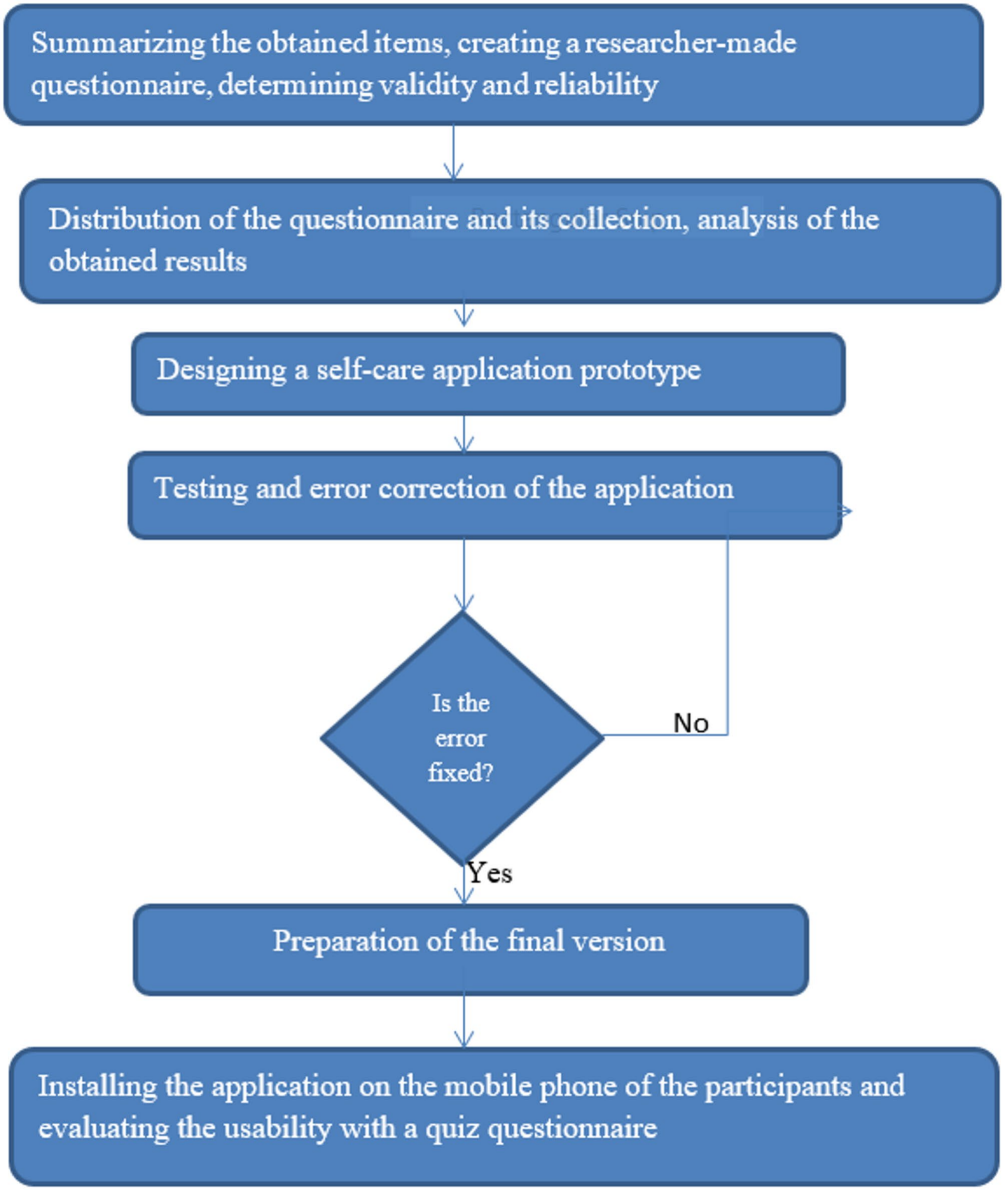
Flow diagram of study

The present study was a developmental-descriptive study conducted in two phases From March 20 to December 10, 2023 (Fig. 1).

### Phase 1: needs assessment for the mobile application

To identify the self-care program requirements, a review of previous studies [22, 25, 31], guidelines [32, 33], and existing applications [22, 25] was conducted. The results were summarized and formulated into a researcher-made questionnaire based on the feedback from the research team. The questionnaire included demographic information of participants (fertility specialists and patients) and information related to the patients’ condition. The educational content and self-care section of the questionnaire comprised the following categories: Patient information (19 items) disease-related data (7 items), Nutrition (22 items), treatments and medications (8 items), physical activity (7 items), personal habits and activities (10 items), sexual history (6 items), and software features (14 items). Additionally, an open-ended question was included at the end of the questionnaire to collect participants’ opinions and suggestions (Supplementary file 1). Each question had two options to determine its importance for inclusion in the mobile application (essential = 1, non-essential = 0). The face validity of the questionnaire was confirmed by four Infertility specialists and four medical informatics experts. Face validity for each questionnaire item was CVI = 0.84.The reliability of the questionnaire was calculated using KR-20, resulting in a coefficient of 0.85.

In the case of open-ended questions, the participants’ suggestions were first reviewed and analyzed by the research team. After being summarized, they were provided to the participants againThe research community included all male patients visiting the Royesh Helal Iran Clinics and Shahid Sadoughi Infertility Center in Yazd within one month. These centers are among the most equipped and well-known in Iran, and their specific geographic locations (one in the capital and the other in the geographical center of Iran) have led to a diverse patient population in terms of ethnicity and culture. The inclusion criteria for patients were being at least 20 years old, owning a smartphone with an Android operating system above version 5, and undergoing treatment for a minimum of 3 month. During the period of permission from the institute, 130 patients were referred, according to the inclusion criteria, 110 people were included in the study, 86 participants (Krejcie and Morgan’s table) were selected through accessible sampling of which only 60 patients completed the questionnaire completely. The total number of Physicians working in the center was 42, of which 38 were randomly selected. But due to Personal and business issues, non-cooperation, travel, only 20 Physicians completed the questionnaire. The inclusion criterion for Physicians was having at least 2 years of work experience. And The Exclusion criteria include lack of access to physicians, individuals’ unwillingness to participate in the study, or incomplete questionnaires. In this study, after obtaining the necessary permissions, two separate researchers simultaneously visited these centers for data collection over a specified one-month period. The research objectives were explained, and participants were provided with oral and written informed consent. The questionnaire was distributed among the participants and collected after completion. The obtained data were analyzed using SPSS software version 21, and descriptive statistical analysis (mean and standard deviation) was reported. Information needs that 70% of participants confirmed as “essential” were considered in the design of the mobile application. In case of contradiction between the views of the two participating groups, a decision is made with the opinion of the consultant and the research team.

### Phase 2: design and evaluation

**Fig. 2:**
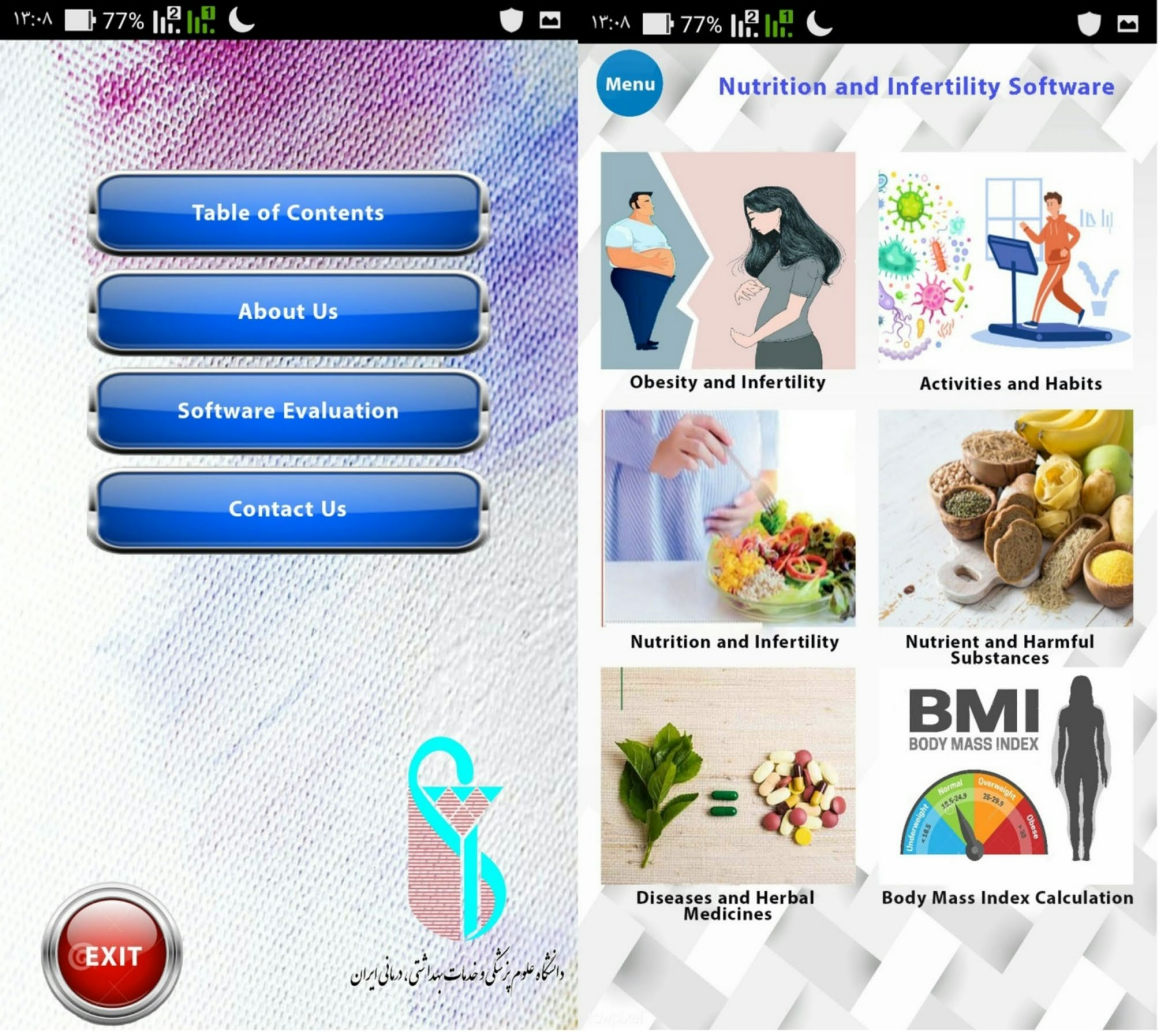
Some pages of application male infertility

In this phase, considering the identified and confirmed needs from the first phase, a self-care mobile application for men experiencing infertility issues was designed. The application was implemented in the Basic4Android (B4A) environment using Java classes (JCL) and the Visual Basic programming language. Some of its pages are depicted in (Fig. 2). After creating the initial version to ensure its proper functionality, the software was randomly installed on several mobile phones. After addressing the issues and receiving final approval from the research team, the final version was prepared. To assess the usability of this application, after obtaining informed consent from the patients verbally and in writing, the application was installed, and necessary instructions were provided orally. it was installed on the mobile phones of 200 patients on October 2023, at the Shahid Sadoughi Infertility Center and the Red Crescent Center. Patients were requested to express their feedback on the usability of the application after two weeks of use. The usability evaluation was conducted using one of the common questionnaires, QUIS (Questionnaire of User Interface Satisfaction) questionnaire version 7, which was designed both in paper format and electronically within the application and provided to the users. Considering the existing conditions, the inclusion criteria for this study involved men aged at least 20 years, undergoing treatment for a minimum of 3 months, and having a smartphone with an Android system version 5 or above. This QUIS questionnaire, based on the 10-point Likert scale zero (the lowest) to nine (the highest).

This questionnaire sections contains “overall reactions to The Software, Screen, Terminology and System Information, Learning, System Capabilities and Usability And User Interface”, each of which contains several sub-sections. The average score of each section was reported as poor (0 -2.99), average (3 -5.99), good (6–9).

## Results

In the first phase of this study, the educational needs of the self-care application for men with infertility problems were collected in the form of a questionnaire.

The demographic information of the participants is presented in Table 1.

**Table 1:** Demographic information of participants

	Variable		Count	Frequency Distribution
Physician	Gender	Male	15	75
		Female	5	25
	Age	40 >	6	30
		40–50	9	45
		50<	5	25
	Work Experience	2–9	10	50
		10–20	6	30
		20<	4	20
Patient	Age	18–24	9	15
		25–31	17	28.3
		32–38	21	35
		38<	13	21.7
	Education Level	Diploma and below	7	11.7
		Above diploma	14	23.3
		Bachelor	23	38.3
		Master or higher	16	26.7
	Treatment Duration	1–6 months	26	43.3
		6–12 months	19	31.7
		12–18 months	8	13.3
		18 months or more	7	11.7

In this study, the majority of participating physicians were men (75%), primarily in the age group of 40 years and above (70%) And its years. Among the participating patients, most were in the age group of 32 years and above (56.7%) and averages for age was 34 and held a bachelor’s degree (65%). The average of Treatment Duration of the patients was 8.4 years.

Participants’ responses regarding essential aspects of the application design, as outlined in the questionnaire, were categorized into sections: patient information (Table 2), disease-related data (Table 3), Nutrition (Table 4), treatments and medications (Table 5), physical activities (Table 6), personal habits and behaviors (Table 7), sexual history (Table 8), and software features (Table 9).

**Table 2:** The frequency of educational needs in the category of “patient information”

Suggested items	Patient		Physician	
	Essential(*n*)	Essential (%)	Essential(*n*)	Essential (%)
first name and last name	56	93.3	20	100
father’s name	20	33.3	8	40
National Code	50	83.3	18	90
Date of birth	44	73.3	17	85
place of birth	34	56.7	12	60
height	60	100	20	100
Weight	60	100	20	100
marital status	15	25	13	65
duration of marriage	55	83.3	20	100
Number of children	44	73.3	15	75
Family history of infertility	52	86.7	20	100
Type of insurance	45	75	15	75
blood type	60	100	20	100
Man’s job	55	91.7	18	90
Woman’s job	46	76.7	16	80
Income level	51	85	16	80
Residential area	49	81.7	15	75
Address of residence	35	58.3	10	50
Phone	52	86.7	20	100

**Table 3:** The frequency of educational needs in the category of “disease-related data”

Suggested items	Patient		Physician	
	Essential(*n*)	Essential (%)	Essential(*n*)	Essential (%)
Definitions (primary infertility, secondary infertility, lifestyle.)	60	100	20	100
Prevalence of infertility	60	100	20	100
Reasons of infertility	60	100	20	100
Signs of infertility	60	100	20	100
Diagnostic methods	60	100	20	100
All kinds of laboratory and genetic tests	60	100	20	100
The impression of chronic diseases (diabetes, etc.)	60	100	20	100

**Table 4:** The frequency of educational needs in the category of “nutrition”

Suggested items	Patient		Physician	
	Essential(*n*)	Essential (%)	Essential(*n*)	Essential (%)
The importance of nutrition	60	100	16	80
treatment regimen	51	85	20	100
Effects of obesity	60	100	20	100
Weight management	58	93.3	20	100
Food habits	52	86.7	18	90
Diets (Mediterranean, Western, etc.)	56	93.3	17	85
Allergy to a certain food	49	91.7	14	70
Meat and fish	45	75	16	80
Antioxidant intake	60	100	20	100
Fruit and vegetable	53	88.3	20	100
supplements and vitamins	60	100	20	100
Micronutrients (iron, zinc, D, etc.)	60	100	20	100
Oils consumption (corn oil, olive oil, sunflower oil)	54	90	18	90
Carbohydrate consumption	60	100	18	90
Protein consumption	60	100	18	90
fast food and ready meals	60	100	19	95
Eating fried foods	56	93.3	17	85
The amount of calories consumed per meal	49	81.7	15	75
Consumption of sugary beverages	26	43.3	13	65
coffee consumption	48	80	15	75
tea consumption	43	71.7	14	70
Amount of sugar and sugar intake	58	96.7	15	75

**Table 5:** The frequency of educational needs in the category of “treatments and medications”

Suggested items	Patient		Physician	
	Essential(*n*)	Essential (%)	Essential(*n*)	Essential (%)
Knowledge of medications	60	100	20	100
The importance of consumption of medications	60	100	20	100
medications combinations	19	31.7	12	60
Conditions of storage and use of medications	60	100	20	100
Complications of medications use	60	100	20	100
Complications of non-use medications	60	100	18	90
Traditional medicine	60	100	14	70
combinations medications interactions	60	100	20	100

**Table 6:** The frequency of educational needs in the category of “physical activities”

Suggested items	Patient		Physician	
	Essential(*n*)	Essential (%)	Essential(*n*)	Essential (%)
importance and impact of exercise	60	100	20	100
Type of activity (light, intense)	36	60	10	50
All kinds of sports	48	80	15	75
The amount of exercise during the day	26	43.3	15	75
The right way to do physical activities	60	100	20	100
suggested time of physical activities	72	70	15	75
Important points in sports	60	100	20	100

**Table 7:** The frequency of educational needs in the category of “personal habits and behaviors”

Suggested items	Patient		Physician	
	Essential(*n*)	Essential (%)	Essential(*n*)	Essential (%)
sleep	36	60	18	90
car driving	18	30	13	65
Use of tobacco	60	100	20	100
Traditional drug use	51	85	18	90
Use of industrial drugs	46	76.7	16	80
Listen to music	60	100	19	95
Use of mobile phones	60	100	15	75
alcoholic beverages	39	65	18	90
The amount of entertainment per week	27	45	11	55
Amount of time spent with family per week	49	81.7	16	80

**Table 8:** The frequency of educational needs in the category of “sexual history”

Suggested items	Patient		Physician	
	Essential(*n*)	Essential (%)	Essential(*n*)	Essential (%)
The role of sexual history	60	100	20	100
amount of semen	53	88.3	20	100
Sperm count	51	85	20	100
sperm Morphology	48	80	20	100
healthy the sperm	60	100	20	100
Sperm quality	60	100	20	100

the number of times of sex, the history of sexually transmitted infections, and the sexual activity are important options

**Table 9:** The frequency of educational needs in the category of “software features”

Suggested items	Patient		Physician	
	Essential(*n*)	Essential (%)	Essential(*n*)	Essential (%)
Calculate BMI	60	100	20	100
physicians visit registration	60	100	18	90
Test registration Laboratory	60	100	16	80
physicians appointment reminder	60	100	20	100
remember to take medicine	55	91.6	20	100
Diet reminder feature	40	66.7	13	65
Laboratory Test time reminder	60	100	15	75
Report physical activity and calorie consumption	52	86.7	18	90
remind exercise	46	76.7	16	80
send educational messages weekly	45	75	20	100
Ability to exchange text messages	39	65	12	60
provide motivational messages	48	80	15	75
Customized for the user	60	100	17	85
show film and animation	57	95	18	90

The results showed that all items in the sections ‘Patient Information’ and ‘Health History (Sexual)’ were approved by the participants. In the ‘Patient Information’ section, out of 19 items, most were deemed essential for both patients and physicians except for ‘Father’s Name,’ ‘Place of Birth,’ ‘Marital Status,’ and ‘Address.’ In the ‘Nutrition’ section, only ‘Consumption of sugary beverages’ out non-essential of 22 items was considered unessential for both patients and physicians. In the ‘Treatment and Medications’ section, the subcategory ‘Drug Combinations’ did not achieve the essential score for both patients and physicians. In the ‘Physical Activities’ section, the item ‘Type of activity (light, intense)’ and in the ‘Personal Habits and Behaviors’ section, items ‘Driving’ and ‘The amount of entertainment per week’ were not approved by the participants. Regarding the ‘Software Features’ section, items ‘Diet reminder feature’ and ‘Ability to exchange text messages’ were not considered essential according to participants’ perspectives. In this study, items that had contradictions in opinions between patients and physicians were decided upon after reconciling the viewpoints and consulting with the research team. Furthermore, participants’ suggestions were examined, and after summarizing and integrating them by the research team, participants’ evaluations were considered, and the results are presented in (Table 10). In this section, out of 12 items, only two items, ‘Introduction to infertility centers’ and ‘Ability to exchange messages with other infertility patients,’ were not deemed essential by both participant groups. There was also a difference of opinion between physicians and patients in items: The duration of the patient’s decision to have a child, Depression management, List of selected physicians, which were considered in the application according to the consultant and the research team.

**Table 10:** Frequency distribution of responses regarding additional suggestions of participants

Suggested items		Patient		Physician	
		Essential(n)	Essential (%)	Essential(n)	Essential (%)
Recommended by physicians	The duration of the patient’s decision to have a child	40	66.7	18	90
	Methods used to prevent pregnancy by the patient	50	83.3	15	75
	Important lifestyle advice	55	91.7	16	80
	Depression management	52	86.6	12	60
Recommended by Patient	Introduction of treatment methods and the benefits	52	86.7	16	80
	Teaching the impact of emotional cases	43	71.7	15	75
	Introduction to infertility centers	37	61.7	13	65
	Introduction of counseling centers	46	76.7	17	85
	List of selected physicians	52	86.7	13	65
	Common disease in infertility and treatment method	47	78.3	15	75
	Ability to exchange messages with other infertile patients	38	63.3	9	45
	Ability to communicate directly between the patient and the physicians	56	93.3	14	70

In the second phase, In B4A software, the application was implemented and the prototype was built. After the approval of the research team, the final version was obtained. Some of its pages are depicted in (Fig. 2).

After that, the Self-Care application was installed on 200 patients’ phones for usability evaluation using the QUIS questionnaire. Participants’ demographic information in the application evaluation is provided in (Table 11).

**Table 11:** Demographic information of participants in the evaluation

Variable		Count	Percentage
Age	18–24	28	14
	25–31	51	25.5
	32–38	69	34.5
	38<	52	26
Duration of Marriage (Years)	1–3	35	17.5
	4–6	84	42
	7–9	63	31.5
	9<	18	9
Education Level	Diploma and below	17	8.5
	Above Diploma	38	19
	Bachelor	54	27
	Master Or Higher	91	45.5
Place Of Residence	Urban	136	68
	Rural	64	32

The results of the usability evaluation of the participants to different parts of the QUIS questionnaire are shown in Table 12.

**Table 12:** The average scores of the participants’ opinions on usability

Scale	Mean	SD
Overall Reactions to The Software	8.01	0.62
Screen	7.5	0.70
Terminology and System Information	7.9	0.94
Learning	8.36	0.37
System Capabilities	7.82	0.86
Usability And User Interface	8.24	0.29
Mean score	7.97	0.63

According to the results, the average usability evaluation score of this application was 7.97 and was analyzed at the “good” level. In this questionnaire, which consists of 6 sections, based on the participants’ views, the highest score was obtained in sections “Learning” and “Usability and User Interface”, with an average score of 8.36 and 8.24, respectively. Also, the lowest score was obtained in section “Screen” with an average of 7.5.

## Discussion

In this study, the information needs of a self-care application for infertile men were prepared in several sections, and based on that, the application was designed, and finally, the usability of the application was evaluated. In this study, all items in the section “disease-related data” which definitions, symptoms, treatment, etc. related to infertility, were deemed essential by all participants, indicating the importance of initial education. Also, in items such as “Allergy to a certain food” and “tea consumption” in section “Nutrition”; item “The amount of exercise during the day” in section “physical activities”; Also, in items such as “sleep”,” alcoholic beverages” in section “personal habits and behaviors”, there was a difference in opinion between the views of physicians and patients, which varies according to the cultural differences of the study participants, economic problems, literacy level, etc.

In the physicians Recommended section, item “The duration of the patient’s decision to have a child” was not considered essential from the patients’ perspective, while physicians considered it essential (90%), because it can be stated that patients think that if they do not conceive after a few months of trying, then they have an infertility problem, and physicians, who are aware of this false belief, considered education on this subject to be very essential. Both groups considered the “Methods used to prevent pregnancy by the patient” and “Important lifestyle advice” essential. “Methods used to prevent pregnancy by the patient” items determine what insight the patient has about his reproductive health and considering that methods of contraception affect the level of male semen quantity, as well as the type and duration of its use have different consequences on the reproductive health of the individual, so this option helps physicians and health care providers for treatment and on the other hand, patients are aware of its effect and avoid unwanted complications in the future. From the patients’ point of view, item “Depression management” was necessary, while the physicians were against it. This is because the patients are under pressure due to family, cultural and economic psychological pressures, which affects the treatment outcome and causes psychological problems such as depression, so they are willing to receive depression management and to increase their knowledge.

In the patient Recommended section: The “Introduction of treatment methods and the benefits”, “Teaching the impact of emotional cases”, “Introduction of counseling centers”, “Common disease in infertility and treatment method” items were deemed essential from the perspective of both groups. These suggested items address factors affecting infertility treatment that have received less attention, while increasing knowledge and impact on the infertility process in men. Patients considered item “List of selected physicians” essential, while physicians considered it unnecessary. It seems that patients consider the role of experienced and skilled physicians in treatment to be very effective.

The results of the study by Hamidzadeh et al. [34] showed that men with infertility problems need education in the field of infertility knowledge, treatments, lifestyle improvement, nutrition, physical activity, etc. Also, in the study by Hesari et al. [35], information needs were classified into 4 main topics with 20 subcategories. Participants also emphasized culturally appropriate education, reasons for treatment failure, time of taking prescribed medications, and psychological education.

In the application designed in the study by Langarizadeh et al. [36], educational items such as scientific definitions of infertility, lifestyle, diet, nutrition, physical activity, and personal habits were included, and the results of the evaluation with the QUIS questionnaire were with an average score of 7.44. The evaluation of the ‘Infotility’ application in the study by Grunberg et al. [37] indicated high participant satisfaction with virtual training and the quality of the training materials. The results of the study by Schick et al. [38] also showed that, given the high stress level and lack of consultants, mobile-based training increased knowledge and impact.

The results of the study by Miner et al. [23] in evaluating a mobile health application in the field of infertility showed that the medical information section (availability of medical centers) received less attention, while the “lifestyle” section received the most attention and received high satisfaction ratings.

Boedt et al. [39] evaluated the MoMiFer-app mobile application in the area of ​​infertility, and the results showed a positive and promising effect on symptoms of emotional distress and overall quality of life. The results of the study by Fusco et al. [26] in evaluating the quality of mobile health applications in the field of male infertility showed that most of these applications focused on diagnosis and treatment. Similarly, Katya Kruglova et al. [25] examined the effectiveness of a mobile application in increasing fertility knowledge specifically for men. The results obtained from knowledge scores before and after the intervention indicated an increase in men’s awareness of identifying infertility risk factors and improved fertility knowledge. In this study, an application was designed and evaluated. The results of the study showed that participants were willing to use the application and found it useful for increasing knowledge and self-care. They also found its use satisfying and effective. Therefore, it seems that the development of this application will increase its use and knowledge.

In this study, all the results of these studies were used, and in order to select the best features and content for this application, various stakeholders (physicians and patients) were used to participate in this study. An attempt was also made to use the features of other patients’ applications in the design. On the other hand, most of the programs in the field of infertility are designed for women. In this study, considering the equal share of women and men in infertility, this application was designed and evaluated specifically for men.

### Limitations

Several limitations of this study should be noted. Firstly, this study was conducted in Persian, the predominant language of Iran, taking into account the cultural norms and customs of the country. However, given the diverse lifestyles and ethnicities across Asia, future research could explore this topic in various languages to assess the impact of different lifestyles and ethnic backgrounds. Secondly, the educational concern addressed in this study specifically targeted men, leading to the design of the application exclusively for them. Additionally, there were challenges related to participants’ cooperation in the study and evaluation. These challenges were overcome by clearly explaining the study’s objectives to the participants and by providing questionnaires in both paper and electronic formats. Furthermore, it is important to note that this study only focused on usability evaluation. In future research, assessments could be expanded to other areas, such as content evaluation and the effectiveness of the designed application, also Exploring the role of the application in increasing awareness and improving useful information literacy could also be a valuable aspect to consider in future studies.

## Conclusion

This study was conducted with the aim of determining self-care needs, designing and evaluating a mobile phone-based application for men with infertility issues. A needs assessment for the application was conducted for men facing infertility issues, taking into account cultural norms, customs, lifestyles, and more. This was achieved through questionnaires, and the participants in the study confirmed the need for such an application. Following the application’s design, usability evaluations were conducted. It is anticipated that the designed application will increase awareness, empower patients in managing their health, enhance communication between patients and healthcare providers, and receive a positive reception from all involved parties in the treatment process. This includes patients, their families, healthcare professionals, and even healthcare policymakers, positioning the application as a valuable tool in the realm of healthcare.

## Supplementary Information

Supplementary Material 1.

## Data Availability

The data used and analysed during the current study are not publicly available due Shahid Sadoughi University of Medical Sciences policy but are available from the corresponding author on reasonable request.

## References

[1] Nadjarzadeh A, Fallahzadeh A, Abasi A, Poornematy MM, Farahzadi HR, Fatemi Aghda SA. Determining the content and needs assessment a mobile-based self-care program in infertile men. BMC Medical Informatics and Decision Making. 2023 13;23(1):258. 37957627 10.1186/s12911-023-02366-2 PMC10644630

[2] Nagórska M, et al. Health related behaviors and life satisfaction in patients undergoing infertility treatment. Int J Environ Res Public Health. 2022;19(15):9188. 35954545 10.3390/ijerph19159188 PMC9367928

[3] Bagi M. Prevalence, reasons and consequences of childlessness in the world and iran: a systematic review. J Popul Assoc Iran. 2023;18(35):97–148.

[4] Barratt CL, et al. A global approach to addressing the policy, research and social challenges of male reproductive health. Hum Reprod Open. 2021;2021(1):hoab009. 33768166 10.1093/hropen/hoab009 PMC7982782

[5] Baker P. Who self-cares wins: an updated perspective on men and self‐care. Trends Urol Men’s Health. 2019;10(3):19–22.

[6] Karimi FZ, et al. Psycho-social effects of male infertility in Iranian women: a qualitative study. Iran J Obstet Gynecol Infertility. 2016;19(10):20–32.

[7] Garrido N, Hervás I. Personalized medicine in infertile men. Urologic Clin. 2020;47(2):245–55. 10.1016/j.ucl.2019.12.011 32272996

[8] Maleki BH, Tartibian B. High-intensity exercise training for improving reproductive function in infertile patients: a randomized controlled trial. J Obstet Gynecol Can. 2017;39(7):545–58. 28625282 10.1016/j.jogc.2017.03.097

[9] Grunberg PH, et al. Infertility patients’ need and preferences for online peer support. Volume 6. Reproductive biomedicine & society online; 2018. pp. 80–9. 10.1016/j.rbms.2018.10.016 PMC6282097 30547107

[10] Bai X, et al. An integrative approach to uncover the components, mechanisms, and functions of traditional Chinese medicine prescriptions on male infertility. Front Pharmacol. 2022;13:794448. 36034828 10.3389/fphar.2022.794448 PMC9403420

[11] Harper JC, et al. The international fertility education initiative: research and action to improve fertility awareness. Hum Reprod Open. 2021;2021(4):hoab031. 34532596 10.1093/hropen/hoab031 PMC8441587

[12] Greil AL, et al. Degrees of medicalization: the case of infertility health-seeking. Sociol Q. 2020;61(2):347–65. 32863442 10.1080/00380253.2019.1625731 PMC7449256

[13] Panda K, Rath SP. Behavioural model based strategies for better adoption of infertility treatment. Med Res Archives. 2023;11(3):1–18

[14] Langarizadeh M, et al. Design and evaluation of an educational mobile program for liver transplant patients. BMC Health Serv Res. 2023;23(1):974. 37684647 10.1186/s12913-023-09989-1 PMC10492268

[15] Barron ML, et al. Fertility health knowledge in US adults: men narrowing the knowledge gap. Am J Men’s Health. 2022;16(5):15579883221117915. 36112813 10.1177/15579883221117915 PMC9478737

[16] Pala D, et al. Smartphone applications for nutrition support: A systematic review of the target outcomes and main functionalities. Int J Med Informatics. 2024;105351:p. 10.1016/j.ijmedinf.2024.105351 38295584

[17] Mortezaei S, et al. Development and usability evaluation of a mHealth application for albinism self-management. BMC Med Inf Decis Mak. 2023;23(1):1–9. 10.1186/s12911-023-02202-7 PMC10262590 37312174

[18] Seyfi Z, et al. Identifying required data elements for designing A Mobile-Based application for Self-Care of women living with endometriosis. Front Health Inf. 2023;12:137.

[19] Sarpourian F, et al. Application of telemedicine in the ambulance for stroke patients: a systematic review. Prehosp Disaster Med. 2023;38(6):774–9. 37877359 10.1017/S1049023X23006519

[20] Kazemnejad A, et al. Therapy-based expert system on function and postural stability after anterior cruciate ligament reconstruction: a pilot study. BMC Musculoskelet Disord. 2023;24(1):617. 37516871 10.1186/s12891-023-06735-w PMC10386671

[21] Samad-Soltani T, Ghanei M, Langarizadeh M. Development of a fuzzy decision support system to determine the severity of obstructive pulmonary in chemical injured victims. Acta Informatica Med. 2015;23(3):138. 10.5455/aim.2015.23.138-141 PMC4499289 26236078

[22] Langarizadeh M, et al. Identifying and validating the educational needs to develop a Celiac Self-Care system. BMC Prim Care. 2023;24(1):121. 37316859 10.1186/s12875-023-02076-8 PMC10265559

[23] Miner SA, et al. Who needs an app? Fertility patients’ use of a novel mobile health app. Digit Health. 2022;8:20552076221102248. 35646384 10.1177/20552076221102248 PMC9131380

[24] Daumler D, et al. Men's knowledge of their own fertility: a population-based survey examining the awareness of factors that are associated with male infertility. Hum Reprod. 2016;31(12):2781–90. 10.1093/humrep/dew265 PMC5193328 27816924

[25] Kruglova K, et al. Risky business: increasing fertility knowledge of men in the general public using the mobile health application infotility XY. Am J Men’s Health. 2021;15(5):15579883211049027. 34697968 10.1177/15579883211049027 PMC8552396

[26] Fusco GM, et al. Male infertility, what mobile health applications know: quality analysis and adherence to European association of urology guidelines. Archivio Italiano Di Urol E Andrologia. 2022;94(4):470–5. 10.4081/aiua.2022.4.470 36576473

[27] Pedro J, et al. What do people know about fertility? A systematic review on fertility awareness and its associated factors. Ups J Med Sci. 2018;123(2):71–81. 29957086 10.1080/03009734.2018.1480186 PMC6055749

[28] Dezhkam L, Darvishi Tafvizi M, Kalani N. Different dimensions of infertility phenomenon in the life of Iranian women: a systematic review study. Iran J Obstet Gynecol Infertility. 2023;26(3):90–108.

[29] Nadjarzadeh A, et al. Determining the content and needs assessment a mobile-based self-care program in infertile men. BMC Med Inf Decis Mak. 2023;23(1):258. 10.1186/s12911-023-02366-2 PMC10644630 37957627

[30] Ezabadi Z, et al. Identification of reproductive education needs of infertile clients undergoing assisted reproduction treatment using assessments of their knowledge and attitude. Int J Fertility Steril. 2017;11(1):20. 10.22074/ijfs.2016.4728 PMC5215707 28367301

[31] Langarizadeh M, et al. The nutritional content required to design an educational application for infertile women. BMC Womens Health. 2023;23(1):22. 36650480 10.1186/s12905-023-02156-y PMC9843656

[32] Infertility TGGoU, et al. Evidence-based guideline: unexplained infertility†. Hum Reprod. 2023;38(10):1881–90. 37599566 10.1093/humrep/dead150 PMC10546081

[33] Lindsay TJ, Vitrikas KR. Evaluation and treatment of infertility. Am Fam Physician. 2015;91(5):308–14. 25822387

[34] Hamidzadeh A, et al. The effect of e-health interventions on meeting the needs of individuals with infertility: a narrative review. Middle East Fertility Soc J. 2023;28(1):1–15. 10.1186/s43043-023-00137-7 PMC10140700 37152275

[35] Hesari ZHNA, et al. The need for a training software among Iranian infertile couples: a qualitative study. Int J Fertility Steril. 2019;13(2):118. 10.22074/ijfs.2019.5727 PMC6500074 31037922

[36] Langarizadeh M, Fatemi Aghda SA, Nadjarzadeh A. Design and evaluation of a mobile-based nutrition education application for infertile women in Iran. BMC Med Inf Decis Mak. 2022;22(1):58. 10.1186/s12911-022-01793-x PMC8894566 35246119

[37] Grunberg PH, et al. Development and evaluation of an online infertility peer supporter training program. Patient Educ Couns. 2020;103(5):1005–12. 31761526 10.1016/j.pec.2019.11.019

[38] Schick M, et al. Smartphone-supported positive adjustment coping intervention (PACI) for couples undergoing fertility treatment: a randomised controlled trial protocol. BMJ Open. 2019;9(7):e025288. 31289056 10.1136/bmjopen-2018-025288 PMC6629398

[39] Boedt T, et al. Protocol: evaluation of a stand-alone mobile mindfulness app in people experiencing infertility: the protocol for an exploratory randomised controlled trial (MoMiFer-RCT). BMJ Open. 2022;12(2):1–7. 10.1136/bmjopen-2021-050088 PMC8811542 35110309

