# Supplementary material for: Design and evaluation of an educational and self-care application for infertile men: perspective of physician and patients
Source: BMC Health Serv Res. 2025 May 19;25:722. doi: 10.1186/s12913-025-12900-9 (PMC12090469; doi:10.1186/s12913-025-12900-9)
Supplement: Supplementary file 1 — Supplementary Material 1. [file 12913_2025_12900_MOESM1_ESM.docx]

Dear respondents,

This questionnaire is designed to identify the necessary items for designing a conceptual model of app for infertile men from the perspective of stakeholders. The questions are organized in different sections and have two “**Essential**” and “**Non-Essential**” answers.

Accurate response will help us to achieve research goals and correct results. The time to answer the questionnaire is about 25 to 30 minutes. If you have any questions, please contact Mr. Seyed Ali Fatemi Aghda. Thank you for your valuable time.

Seyed Ali Fatemi Aghda

Senior expert in health information technology

Email: [afatamy@yahoo.com](mailto:afatamy@yahoo.com)

**A: Demographic Information**

**A.1: Physician:**

**Gender:** Male Female

**Age:** <40 40-50 >50

**Work Experience:** <10 10-20 >20

**A.2: Patient:**

**Age:** 18-24 25-31 32-38 >38

**Education Level**: Diploma and below above diploma Bachelor Master or higher

**Treatment Duration**: 1 - 6 months 6 - 12 months 12 - 18 months 18 months or more

B: Data Requirements items

B.1: Demographic Information

| Row | Information requirements | Essential | Non-Essential |
| --- | --- | --- | --- |
| 1 | First Name And Last Name |  |  |
| 2 | Father's Name |  |  |
| 3 | National Code |  |  |
| 4 | Date Of Birth |  |  |
| 5 | Place Of Birth |  |  |
| 6 | Height |  |  |
| 7 | Weight |  |  |
| 8 | Marital Status |  |  |
| 9 | Duration Of Marriage |  |  |
| 10 | Number Of Children |  |  |
| 11 | Family History Of Infertility |  |  |
| 12 | Type Of Insurance |  |  |
| 13 | Blood Type |  |  |
| 14 | Own Job |  |  |
| 15 | Wife's Job |  |  |
| 16 | Income Level |  |  |
| 17 | Residential Area |  |  |
| 18 | Address |  |  |
| 19 | Phone |  |  |

B.2: Disease-Related Data

| Row | Information requirements | Essential | Non-Essential |
| --- | --- | --- | --- |
| 20 | Definitions (Primary, Secondary, Lifestyle, Etc.) |  |  |
| 21 | Prevalence |  |  |
| 22 | Reasons |  |  |
| 23 | Signs |  |  |
| 24 | Diagnostic Methods |  |  |
| 25 | All Kinds Of Laboratory And Genetic Tests |  |  |
| 26 | The Impression Of Chronic Diseases (Diabetes, Etc.) |  |  |

B.3: Nutrition

| Row | Information Requirements | Essential | Non-Essential |
| --- | --- | --- | --- |
| 27 | The Importance Of Nutrition |  |  |
| 28 | Treatment Regimen |  |  |
| 29 | Effects Of Obesity |  |  |
| 30 | Weight Management |  |  |
| 31 | Food Habits |  |  |
| 32 | Diets (Mediterranean, Western, Etc.) |  |  |
| 33 | Allergy To A Certain Food |  |  |
| 34 | Meat And Fish |  |  |
| 35 | Antioxidant Intake |  |  |
| 36 | Fruit And Vegetable |  |  |
| 37 | Supplements And Vitamins |  |  |
| 38 | Micronutrients (Iron, Zinc, D, Etc.) |  |  |
| 39 | Oils (Corn Oil, Olive Oil, Sunflower Oil) |  |  |
| 40 | Carbohydrate Intake |  |  |
| 41 | Protein |  |  |
| 42 | Fast Food And Ready Meals |  |  |
| 43 | Eating Fried Foods |  |  |
| 44 | The Amount Of Calories Consumed Per Meal |  |  |
| 45 | Consumption Of Sugary Beverages |  |  |
| 46 | Coffee Consumption |  |  |
| 47 | Tea Consumption |  |  |
| 48 | Amount Of Sugar And Sugar Intake |  |  |

B.4: Treatments and Medications

| Row | Information Requirements | Essential | Non-Essential |
| --- | --- | --- | --- |
| 49 | Knowledge Of Drugs |  |  |
| 50 | The Importance Of Consumption |  |  |
| 51 | Drug Combinations |  |  |
| 52 | Conditions Of Storage And Use Of Medicine |  |  |
| 53 | Complications Of Their Use |  |  |
| 54 | Complications Of Non-Use |  |  |
| 55 | Traditional Medicine |  |  |
| 56 | Drug Interactions |  |  |

B.5: Physical Activities

| Row | Information requirements | Essential | Non-Essential |
| --- | --- | --- | --- |
| 57 | Importance And Impact Of Exercise |  |  |
| 58 | Type Of Activity (Light, Intense) |  |  |
| 59 | All Kinds Of Sports |  |  |
| 60 | The Amount Of Exercise During The Day |  |  |
| 61 | The Right Way To Do It |  |  |
| 62 | Suggested Time |  |  |
| 63 | Important Points In Sports |  |  |

B.6: Personal Habits and Behaviors

| Row | Information requirements | Essential | Non-Essential |
| --- | --- | --- | --- |
| 64 | Sleep |  |  |
| 65 | Driving |  |  |
| 66 | Use Of Tobacco |  |  |
| 67 | Traditional Drug Use |  |  |
| 68 | Use Of Industrial Drugs |  |  |
| 69 | Listen To Music |  |  |
| 70 | Use Of Mobile Phones |  |  |
| 71 | Alcoholic Beverages |  |  |
| 72 | The Amount Of Entertainment Per Week |  |  |
| 73 | Amount Of Time Spent With Family Per Week |  |  |

B.7: sexual history

| Row | Information requirements | Essential | Non-Essential |
| --- | --- | --- | --- |
| 74 | The Role Of Sexual History |  |  |
| 75 | Amount Of Semen |  |  |
| 76 | Sperm Count |  |  |
| 77 | Sperm Morphology |  |  |
| 78 | Healthy The Sperm |  |  |
| 79 | Sperm Quality |  |  |

B.8: software features

| .Row | Information requirements | Essential | Non-Essential |
| --- | --- | --- | --- |
| 80 | Calculate BMI |  |  |
| 81 | Doctor's Visit Registration |  |  |
| 82 | Test Registration |  |  |
| 83 | Doctor Appointment Reminder |  |  |
| 84 | Remember To Take Medicine |  |  |
| 85 | Diet Reminder Feature |  |  |
| 86 | Test Time Reminder |  |  |
| 87 | Report Physical Activity And Calorie Consumption |  |  |
| 88 | Remind Exercise |  |  |
| 89 | Send Educational Messages Weekly |  |  |
| 90 | Ability To Exchange Text Messages |  |  |
| 91 | Provide Motivational Messages |  |  |
| 92 | Customized For The User |  |  |
| 93 | Short Film And Animation |  |  |

C:Please enter your suggestions below

………………………………………………………………………………………………………………………………………………………………………………………………………………………………………………………………………………………………………………………………………………
